# Decline in adaptive immune responses within human leptomeningesduring Alzheimer's Disease progression

**DOI:** 10.1002/alz70855_102245

**Published:** 2025-12-23

**Authors:** Katherine M Sheu, Mitchell H Murdock, Na Sun, Manolis Kellis, Li‐Huei Tsai

**Affiliations:** ^1^ Massachusetts Institute of Technology, Cambridge, MA, USA; ^2^ Picower Institute for Learning and Memory, Cambridge, MA, USA

## Abstract

**Background:**

The meninges comprise several layers of connective tissue that regulate the influx and efflux of fluid, protein, and immune cells to and from the brain parenchyma. However, it remains unclear how different cell types within the meninges contribute to the increasing dysfunction of this neurovascular‐immune interface during Alzheimer's Disease (AD) progression.

**Method:**

Here, we profile single nuclei transcriptomes of leptomeninges from 51 individuals with varying degrees of Alzheimer's pathology.

**Result:**

We identify fibroblasts, cell types of the meningeal vasculature, including endothelial cells and smooth muscle cells, and immune cells such as macrophages and lymphocytes. We find that increased global AD pathology is associated with endothelial cell and macrophage downregulation of gene programs associated with antigen presentation. Analysis of matched samples from the prefrontal cortex of a subset of 22 patients show a concordant pattern of decrease in antigen presentation programs in individuals with the greatest amyloid/tau burden.

**Conclusion:**

Our findings point to a concomitant leptomeningeal and brain parenchymal dysfunction of humoral immunity associated with worsened AD pathology, suggesting a target for systemic immune therapy.

